# *N*-(3-Bromo-2-methyl­phen­yl)-2-oxo-1,2-di­hydro­pyridine-3-carboxamide

**DOI:** 10.1107/S1600536812011294

**Published:** 2012-03-21

**Authors:** Yun-Hua Xu, Sihui Long

**Affiliations:** a School of Science, Beijing Jiaotong University, Beijing 100044, People’s Republic of China; b College of Pharmacy, Ohio State University, Columbus, OH 43210, USA

## Abstract

The title compound, C_13_H_11_BrN_2_O_2_, consists of two six-membered rings linked by an amide group and adopts a near planar conformation. The dihedral angle between the two rings is 8.38 (11)°. In the crystal structure, there are intra- and inter­molecular N—H⋯O hydrogen bonds, the latter forming inversion dimers.

## Related literature

For a related structure, see: Long *et al.* (2006 ▶). For background and details of synthesis, see: Ting *et al.* (1990 ▶).
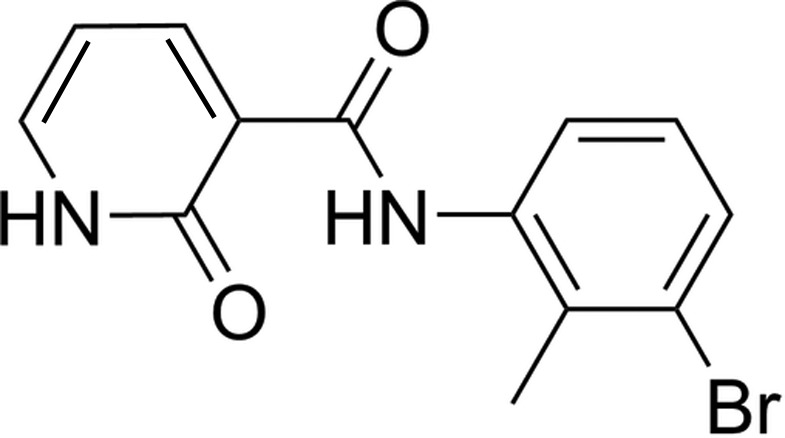


## Experimental

### 

#### Crystal data

C_13_H_11_BrN_2_O_2_
*M* *_r_* = 307.15
Triclinic, 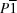

*a* = 7.164 (1) Å
*b* = 7.715 (1) Å
*c* = 10.446 (2) Å
α = 88.23 (1)°
β = 89.18 (1)°
γ = 89.68 (1)°
*V* = 577.01 (16) Å^3^
*Z* = 2
Mo *K*α radiation
μ = 3.56 mm^−1^
*T* = 90 K
0.30 × 0.10 × 0.04 mm

#### Data collection

Nonius KappaCCD diffractometer
Absorption correction: multi-scan (*SCALEPACK*; Otwinowski & Minor, 1997 ▶) *T*_min_ = 0.415, *T*_max_ = 0.871
5027 measured reflections
2637 independent reflections
2273 reflections with *I* > 2σ(*I*)
*R*_int_ = 0.032

#### Refinement

*R*[*F* ^2^ > 2σ(*F* ^2^)] = 0.031
*wR*(*F* ^2^) = 0.072
*S* = 1.07
2637 reflections
164 parameters
H-atom parameters constrained
Δρ_max_ = 0.94 e Å^−3^
Δρ_min_ = −0.61 e Å^−3^

### 

Data collection: *COLLECT* (Nonius, 2002 ▶); cell refinement: *DENZO-SMN* (Otwinowski & Minor, 1997 ▶); data reduction: *DENZO-SMN*; program(s) used to solve structure: *SHELXS97* (Sheldrick, 2008 ▶); program(s) used to refine structure: *SHELXL97* (Sheldrick, 2008 ▶); molecular graphics: *SHELXTL* (Sheldrick, 2008 ▶); software used to prepare material for publication: *SHELXL97*.

## Supplementary Material

Crystal structure: contains datablock(s) global, I. DOI: 10.1107/S1600536812011294/ff2058sup1.cif

Structure factors: contains datablock(s) I. DOI: 10.1107/S1600536812011294/ff2058Isup2.hkl

Supplementary material file. DOI: 10.1107/S1600536812011294/ff2058Isup3.cml

Additional supplementary materials:  crystallographic information; 3D view; checkCIF report

## Figures and Tables

**Table 1:** Hydrogen-bond geometry (Å, °)

*D*—H⋯*A*	*D*—H	H⋯*A*	*D*⋯*A*	*D*—H⋯*A*
N1—H1⋯O2	0.88	1.90	2.660 (3)	144
N2—H2*A*⋯O2^i^	0.88	1.91	2.785 (3)	171

Symmetry code: (i) 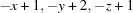.

**Fig. 1.**
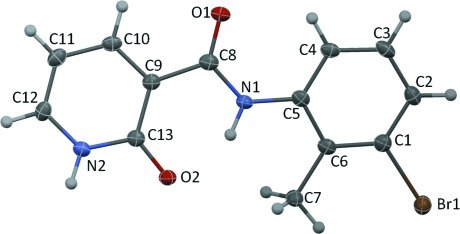
The molecular structure of (I), with displacement ellipsoids drawn at the 50% probability level (arbitrary spheres for the H atoms).

**Fig. 2.**
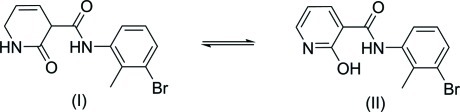
Tautomerism of the title compound.

## supplementary crystallographic information

### Comment

The title compound (I) was obtained as a by-product during an effort to make 2-(2-methyl-3-bromoanilino)nicotinic acid by reacting 2-chloronicotinic acid with 3-bromo-2-methylaniline through a modified procedure from Ting *et al.* (1990). Similar to the case of *N*-(3-chloro-2-methylphenyl)-1,2-dihydro-2-oxo-3 -pyridinecarboxamide (Long *et al.*, 2006), the crystal structure analysis revealed it is the keto-amine (or lactam) tautomer, rather than the hydroxy-pyridine tautomer (II) (Fig. 1, Table 1). The two aromatic rings of the molecule are linked by an amide group. Due to the extended π-conjugation system throughout the whole molecule *via* the amide bridge, the molecule takes a near planar conformation. The dihedral angle between the two aromatic rings is 8.38 (11)°.

Centrosymmetric dimers are formed through intra- and intermolecular N—H···O hydrogen bonds (Table 2). Essentially, the title compound is isostructural with *N*-(3-chloro-2-methylphenyl)-1,2-dihydro-2-oxo-3-pyridinecarboxamide, since the only difference is bromine in the title compound and chlorine in the counterpart.

### Experimental

2-Chloronicotinic acid (1.9 g, 12.1 mmol), 3-bromo-2-methyl-aniline (2.5 g, 13.4 mmol), and pyridine (1.0 ml, 12 mmol) were added to a round-bottom flask, followed by introduction of *p*-toluenesulfonic acid (0.3 g, 1.8 mmol) in 10 ml of water. The resulted solution was refluxed overnight. Colorless solid precipitated out after the mixture was cooled down to room temperature, and it was characterized by NMR to be the title compound (I). Crystals were grown from MeOH solution by slow evaporation.

### Refinement

H atoms were located in difference Fourier maps and subsequently placed in idealized positions with constrained C—H distances of 0.95 (C_Ar_—H), 0.98 (C_Me_—H) and 0.88 Å (N—H). *U*_iso_(H) values were set to 1.2*U*_eq_(C,*N*) or 1.5*U*_eq_(C) for methyl group.

### Crystal data

**Table:**

C_13_H_11_BrN_2_O_2_	*Z* = 2
*M**_r_* = 307.15	*F*(000) = 308
Triclinic, *P*1	*D*_x_ = 1.768 Mg m^−^^3^
Hall symbol: -P 1	Mo *K*α radiation, λ = 0.71073 Å
*a* = 7.164 (1) Å	Cell parameters from 2552 reflections
*b* = 7.715 (1) Å	θ = 1.0–27.5°
*c* = 10.446 (2) Å	µ = 3.56 mm^−^^1^
α = 88.23 (1)°	*T* = 90 K
β = 89.18 (1)°	Thick plate, colourless
γ = 89.68 (1)°	0.30 × 0.10 × 0.04 mm
*V* = 577.01 (16) Å^3^	

### Data collection

**Table:**

Nonius KappaCCD diffractometer	2637 independent reflections
Radiation source: fine-focus sealed tube	2273 reflections with *I* > 2σ(*I*)
Graphite monochromator	*R*_int_ = 0.032
Detector resolution: 18 pixels mm^-1^	θ_max_ = 27.5°, θ_min_ = 2.0°
ω scans at fixed χ = 55°	*h* = −9→9
Absorption correction: multi-scan (*SCALEPACK*; Otwinowski & Minor, 1997)	*k* = −10→10
*T*_min_ = 0.415, *T*_max_ = 0.871	*l* = −13→13
5027 measured reflections	

### Refinement

**Table:**

Refinement on *F*^2^	Primary atom site location: structure-invariant direct methods
Least-squares matrix: full	Secondary atom site location: difference Fourier map
*R*[*F*^2^ > 2σ(*F*^2^)] = 0.031	Hydrogen site location: inferred from neighbouring sites
*wR*(*F*^2^) = 0.072	H-atom parameters constrained
*S* = 1.07	*w* = 1/[σ^2^(*F*_o_^2^) + (0.0231*P*)^2^ + 0.5699*P*] where *P* = (*F*_o_^2^ + 2*F*_c_^2^)/3
2637 reflections	(Δ/σ)_max_ = 0.001
164 parameters	Δρ_max_ = 0.94 e Å^−^^3^
0 restraints	Δρ_min_ = −0.61 e Å^−^^3^

### Special details

**Table:**

Geometry. All e.s.d.'s (except the e.s.d. in the dihedral angle between two l.s. planes) are estimated using the full covariance matrix. The cell e.s.d.'s are taken into account individually in the estimation of e.s.d.'s in distances, angles and torsion angles; correlations between e.s.d.'s in cell parameters are only used when they are defined by crystal symmetry. An approximate (isotropic) treatment of cell e.s.d.'s is used for estimating e.s.d.'s involving l.s. planes.
Refinement. Refinement of *F*^2^ against ALL reflections. The weighted *R*-factor *wR* and goodness of fit *S* are based on *F*^2^, conventional *R*-factors *R* are based on *F*, with *F* set to zero for negative *F*^2^. The threshold expression of *F*^2^ > σ(*F*^2^) is used only for calculating *R*-factors(gt) *etc*. and is not relevant to the choice of reflections for refinement. *R*-factors based on *F*^2^ are statistically about twice as large as those based on *F*, and *R*- factors based on ALL data will be even larger.

### Fractional atomic coordinates and isotropic or equivalent isotropic displacement parameters (Å^2^)

**Table:**

	*x*	*y*	*z*	*U*_iso_*/*U*_eq_	
C1	0.1649 (3)	0.2509 (3)	0.8354 (2)	0.0184 (5)	
C2	0.1129 (3)	0.0867 (3)	0.7987 (3)	0.0194 (5)	
H2	0.0843	−0.0021	0.8608	0.023*	
C3	0.1039 (3)	0.0564 (3)	0.6692 (3)	0.0201 (5)	
H3	0.0677	−0.0547	0.6421	0.024*	
C4	0.1469 (3)	0.1848 (3)	0.5786 (2)	0.0179 (5)	
H4	0.1410	0.1614	0.4900	0.021*	
C5	0.1990 (3)	0.3492 (3)	0.6174 (2)	0.0170 (5)	
C6	0.2097 (3)	0.3859 (3)	0.7489 (2)	0.0160 (5)	
C7	0.2664 (4)	0.5641 (3)	0.7890 (2)	0.0193 (5)	
H7A	0.2662	0.5674	0.8827	0.029*	
H7B	0.1780	0.6505	0.7548	0.029*	
H7C	0.3920	0.5900	0.7555	0.029*	
C8	0.2416 (3)	0.4880 (3)	0.3990 (2)	0.0176 (5)	
C9	0.3033 (3)	0.6573 (3)	0.3379 (2)	0.0161 (5)	
C10	0.2884 (3)	0.6775 (3)	0.2073 (2)	0.0189 (5)	
H10	0.2405	0.5845	0.1603	0.023*	
C11	0.3414 (4)	0.8303 (3)	0.1408 (2)	0.0213 (6)	
H11	0.3299	0.8417	0.0505	0.026*	
C12	0.4098 (4)	0.9616 (4)	0.2098 (2)	0.0199 (5)	
H12	0.4471	1.0668	0.1674	0.024*	
C13	0.3763 (3)	0.7979 (3)	0.4092 (2)	0.0160 (5)	
N1	0.2464 (3)	0.4838 (3)	0.52988 (19)	0.0163 (4)	
H1	0.2850	0.5797	0.5642	0.020*	
N2	0.4252 (3)	0.9437 (3)	0.3384 (2)	0.0174 (4)	
H2A	0.4699	1.0318	0.3796	0.021*	
O1	0.1914 (3)	0.3676 (2)	0.33371 (17)	0.0226 (4)	
O2	0.3985 (2)	0.7976 (2)	0.52859 (16)	0.0190 (4)	
Br1	0.17131 (4)	0.28503 (3)	1.01565 (2)	0.02315 (10)	

### Atomic displacement parameters (Å^2^)

**Table:**

	*U*^11^	*U*^22^	*U*^33^	*U*^12^	*U*^13^	*U*^23^
C1	0.0152 (13)	0.0185 (13)	0.0217 (13)	0.0025 (10)	−0.0020 (10)	−0.0010 (10)
C2	0.0146 (13)	0.0158 (12)	0.0275 (13)	0.0003 (10)	−0.0007 (10)	0.0019 (10)
C3	0.0145 (13)	0.0144 (12)	0.0316 (14)	−0.0011 (10)	−0.0022 (11)	−0.0040 (11)
C4	0.0142 (12)	0.0184 (12)	0.0214 (13)	−0.0003 (10)	−0.0024 (10)	−0.0048 (10)
C5	0.0103 (12)	0.0172 (12)	0.0234 (13)	0.0008 (9)	−0.0019 (10)	−0.0012 (10)
C6	0.0099 (12)	0.0162 (12)	0.0220 (12)	0.0017 (9)	−0.0008 (10)	−0.0035 (10)
C7	0.0238 (14)	0.0165 (12)	0.0178 (12)	−0.0005 (10)	0.0017 (10)	−0.0026 (10)
C8	0.0120 (12)	0.0189 (12)	0.0223 (13)	0.0025 (10)	−0.0013 (10)	−0.0046 (10)
C9	0.0113 (12)	0.0186 (12)	0.0185 (12)	0.0018 (9)	−0.0005 (9)	−0.0021 (10)
C10	0.0149 (13)	0.0224 (13)	0.0198 (12)	0.0006 (10)	−0.0025 (10)	−0.0055 (10)
C11	0.0186 (13)	0.0275 (14)	0.0177 (12)	0.0031 (11)	−0.0024 (10)	−0.0001 (11)
C12	0.0159 (13)	0.0248 (14)	0.0187 (12)	0.0001 (10)	0.0004 (10)	0.0037 (10)
C13	0.0120 (12)	0.0174 (12)	0.0186 (12)	0.0026 (9)	0.0008 (9)	−0.0025 (10)
N1	0.0179 (11)	0.0150 (10)	0.0163 (10)	−0.0017 (8)	−0.0014 (8)	−0.0038 (8)
N2	0.0165 (11)	0.0171 (10)	0.0187 (10)	−0.0014 (8)	−0.0011 (8)	−0.0024 (8)
O1	0.0279 (10)	0.0191 (9)	0.0212 (9)	−0.0042 (8)	−0.0045 (8)	−0.0050 (7)
O2	0.0217 (9)	0.0193 (9)	0.0163 (9)	−0.0035 (7)	−0.0004 (7)	−0.0026 (7)
Br1	0.02871 (16)	0.02031 (14)	0.02034 (14)	0.00082 (10)	0.00016 (10)	0.00063 (10)

### Geometric parameters (Å, º)

**Table:**

C1—C2	1.389 (4)	C8—O1	1.228 (3)
C1—C6	1.394 (4)	C8—N1	1.368 (3)
C1—Br1	1.911 (3)	C8—C9	1.502 (3)
C2—C3	1.382 (4)	C9—C10	1.375 (3)
C2—H2	0.9500	C9—C13	1.439 (3)
C3—C4	1.382 (4)	C10—C11	1.401 (4)
C3—H3	0.9500	C10—H10	0.9500
C4—C5	1.398 (3)	C11—C12	1.359 (4)
C4—H4	0.9500	C11—H11	0.9500
C5—N1	1.403 (3)	C12—N2	1.353 (3)
C5—C6	1.414 (3)	C12—H12	0.9500
C6—C7	1.509 (3)	C13—O2	1.259 (3)
C7—H7A	0.9800	C13—N2	1.371 (3)
C7—H7B	0.9800	N1—H1	0.8800
C7—H7C	0.9800	N2—H2A	0.8800
			
C2—C1—C6	123.6 (2)	O1—C8—N1	124.9 (2)
C2—C1—Br1	115.94 (19)	O1—C8—C9	121.1 (2)
C6—C1—Br1	120.48 (19)	N1—C8—C9	114.0 (2)
C3—C2—C1	118.1 (2)	C10—C9—C13	118.9 (2)
C3—C2—H2	121.0	C10—C9—C8	117.7 (2)
C1—C2—H2	121.0	C13—C9—C8	123.4 (2)
C4—C3—C2	121.1 (2)	C9—C10—C11	122.3 (2)
C4—C3—H3	119.4	C9—C10—H10	118.8
C2—C3—H3	119.4	C11—C10—H10	118.8
C3—C4—C5	119.9 (2)	C12—C11—C10	117.9 (2)
C3—C4—H4	120.0	C12—C11—H11	121.1
C5—C4—H4	120.0	C10—C11—H11	121.1
C4—C5—N1	122.4 (2)	N2—C12—C11	120.5 (2)
C4—C5—C6	120.8 (2)	N2—C12—H12	119.8
N1—C5—C6	116.7 (2)	C11—C12—H12	119.8
C1—C6—C5	116.5 (2)	O2—C13—N2	118.3 (2)
C1—C6—C7	123.5 (2)	O2—C13—C9	125.9 (2)
C5—C6—C7	120.1 (2)	N2—C13—C9	115.8 (2)
C6—C7—H7A	109.5	C8—N1—C5	129.5 (2)
C6—C7—H7B	109.5	C8—N1—H1	115.2
H7A—C7—H7B	109.5	C5—N1—H1	115.2
C6—C7—H7C	109.5	C12—N2—C13	124.7 (2)
H7A—C7—H7C	109.5	C12—N2—H2A	117.7
H7B—C7—H7C	109.5	C13—N2—H2A	117.7
			
C6—C1—C2—C3	−0.5 (4)	N1—C8—C9—C13	5.0 (3)
Br1—C1—C2—C3	179.22 (18)	C13—C9—C10—C11	−0.1 (4)
C1—C2—C3—C4	0.5 (4)	C8—C9—C10—C11	179.8 (2)
C2—C3—C4—C5	−0.5 (4)	C9—C10—C11—C12	0.1 (4)
C3—C4—C5—N1	179.1 (2)	C10—C11—C12—N2	−0.1 (4)
C3—C4—C5—C6	0.4 (4)	C10—C9—C13—O2	−179.7 (2)
C2—C1—C6—C5	0.4 (4)	C8—C9—C13—O2	0.4 (4)
Br1—C1—C6—C5	−179.30 (17)	C10—C9—C13—N2	0.2 (3)
C2—C1—C6—C7	−179.9 (2)	C8—C9—C13—N2	−179.7 (2)
Br1—C1—C6—C7	0.4 (3)	O1—C8—N1—C5	0.3 (4)
C4—C5—C6—C1	−0.4 (3)	C9—C8—N1—C5	179.9 (2)
N1—C5—C6—C1	−179.1 (2)	C4—C5—N1—C8	4.3 (4)
C4—C5—C6—C7	179.9 (2)	C6—C5—N1—C8	−177.0 (2)
N1—C5—C6—C7	1.1 (3)	C11—C12—N2—C13	0.2 (4)
O1—C8—C9—C10	4.8 (4)	O2—C13—N2—C12	179.7 (2)
N1—C8—C9—C10	−174.9 (2)	C9—C13—N2—C12	−0.2 (3)
O1—C8—C9—C13	−175.3 (2)		

### Hydrogen-bond geometry (Å, º)

**Table:**

*D*—H···*A*	*D*—H	H···*A*	*D*···*A*	*D*—H···*A*
N1—H1···O2	0.88	1.90	2.660 (3)	144
N2—H2*A*···O2^i^	0.88	1.91	2.785 (3)	171

Symmetry code: (i) −*x*+1, −*y*+2, −*z*+1.
